# Adjunctive Use of Intravenous Antibiotic Regional Limb Perfusion in Three Cranes with Distal Limb Infections

**DOI:** 10.3390/ani11092673

**Published:** 2021-09-12

**Authors:** Gail L. Huckins, Richard R. Sim, Barry Hartup

**Affiliations:** 1 Department of Surgical Sciences, School of Veterinary Medicine, University of Wisconsin-Madison, Madison, WI 53706, USA; hartup@savingcranes.org; 2 Veterinary Medical Center, Oregon Zoo, Portland, OR 97221, USA; rich.r.sim@gmail.com; 3 Department of Conservation Medicine, International Crane Foundation, Baraboo, WI 53913, USA

**Keywords:** cranes, gruiformes, regional limb perfusion

## Abstract

**Simple Summary:**

Foot and limb infections are common issues in captive cranes, and traditional antibiotic therapy can be unrewarding. Three cranes at two captive institutions in the United States with foot and limb infections were treated with regional limb perfusion of intravenous antibiotics. The details of treatment are described in this paper. All three cranes recovered from their infections following regional limb perfusion in combination with systemic antibiotics.

**Abstract:**

Pododermatitis and wing lesions are commonly reported issues in captive crane species. Regional limb perfusion has been used as a treatment for distal limb infections in several avian species, as systemic antibiotic therapy is often prolonged and unrewarding. A black-necked crane (*Grus nigricollis*), Siberian crane (*Leucogeranus leucogeranus*), and wattled crane (*Bugeranus carunculatus*) were treated with amikacin (5–10 mg/kg IV infusion) regional limb perfusion for cellulitis and osteomyelitis of hind limb digits and alular osteomyelitis and septic arthritis of the carpus, respectively, with a range of 1–3 treatments per case. Clinical signs of infection resolved within 10–40 days following regional limb perfusion combined with oral or parental antibiotic therapy. No side effects were observed following regional limb perfusion. This is the first report of regional limb perfusion in cranes and the first report of intravenous regional limb perfusion in a wing of an avian species.

## 1. Introduction

Pododermatitis and traumatic wing injuries are common causes of morbidity in captive cranes and can lead to bacterial disease of distal tissues [1]. Regional limb perfusion (RLP) with antibiotics is a commonly used technique in human and equine medicine to treat distal limb infections, but its use in avian medicine is rare [2,3]. This technique has previously been described in brown pelicans (*Pelecanus occidentalis californicus*), a loon (*Gavia pacifica*), and a chicken (*Gallus gallus*) [4,5]. The use of ceftiofur with intravenous RLP has been validated in chickens, and a recent pilot study evaluated the use of intraosseous RLP regional limb perfusion in chicken wings [6,7]. Regional limb perfusion allows much higher antibiotic concentrations at infection sites by creating a concentration and pressure gradient between intravascular and extravascular spaces, maintaining low systemic drug levels, and allowing for a decreased frequency of administration, leading to reduced restraint, which is advantageous in avian species [2,3]. This report describes the use of intravenous antibiotic RLP to treat distal limb cellulitis, osteomyelitis, and septic arthritis in three different crane species, including the first use of intravenous RLP in a wing.

## 2. History and Case Presentation

**Figure 1:**
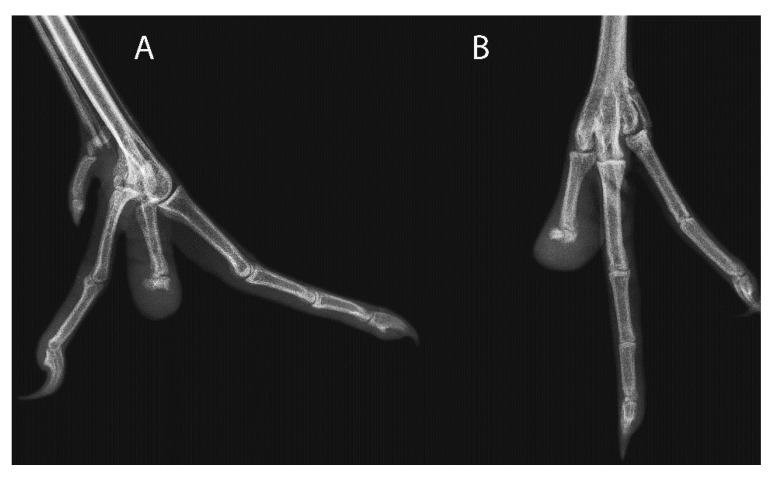
(**A)** Lateral and (**B**) dorsoplantar radiographs of the left foot of Case 1, a 13-year-old female black-necked crane (*Grus nigricollis*) with cellulitis and probable osteomyelitis of the fourth digit of the left foot at time of presentation.

Case 1, a 13-year-old female black-necked crane (*Grus nigricollis*), was presented for a traumatic open luxation of the interphalangeal joint between the second and third phalanges of the left fourth digit. The luxation was initially managed with external coaptation and the bird was treated empirically with ceftiofur crystalline free acid (Excede, Zoetis Inc., Madison, NJ 07940, USA; 10 mg/kg IM q3d for 30 days) and meloxicam (Boehringer Ingelheim Animal Health USA Inc., Duluth, GA 30096, USA; 0.5 mg/kg IM q48h for 30 days). The digit became edematous approximately on day 10 and clindamycin (Ohm Laboratories Inc., New Brunswick, NJ 08901, USA; 100 mg/kg PO q24h for 10 days) was added. On day 13, the underlying medial tendon and second phalangeal bone were visibly necrotic, and the toe was amputated at the level of the proximal second phalanx under general anesthesia. On day 27, the amputation site was erythematous, swollen, and warm to the touch, and radiographs of the left distal limb showed soft tissue swelling and bone lysis of the remaining distal digit (Figure 1). A CBC revealed a leukocytosis (39,700/µL) characterized by a heterophilia (17,070/µL) [8]. Given the concern for cellulitis and osteomyelitis, RLP with antibiotics was elected. A 23-ga butterfly catheter as placed in the left medial metatarsal vein and a 1 cm diameter penrose drain was wrapped once around the left tibiotarsus, then secured with hemostats proximal to the tarsal joint as a tourniquet. Amikacin (sterile amikacin sulfate solution, 50 mg/mL; Wedgewood Pharmacy, Swedesboro, NJ 08085, USA; 11.5 mg/kg IV) was infused through the catheter and the tourniquet was left in place for 15 min. A bandage of a cotton ball and 2.5 cm conforming self-adhering bandage (Vetrap, 3M, St. Paul, MN 55144, USA) was placed for 3 min after the butterfly catheter was removed. The bird was started on enrofloxacin (Baytril, Bayer Corp, Shawnee, KS, 66216, USA; 13 mg/kg q24h SQ for one dose and PO for 9 days). On day 36, the digit was swollen, but no longer warm to the touch and the bird had no discomfort on palpation. A repeat complete blood count showed improvement of the previously noted leukocytosis (25,300/µL). Radiographs were not repeated, but this bird has shown no lameness or evidence of infection for 4 years since the RLP procedure.

**Figure 2:**
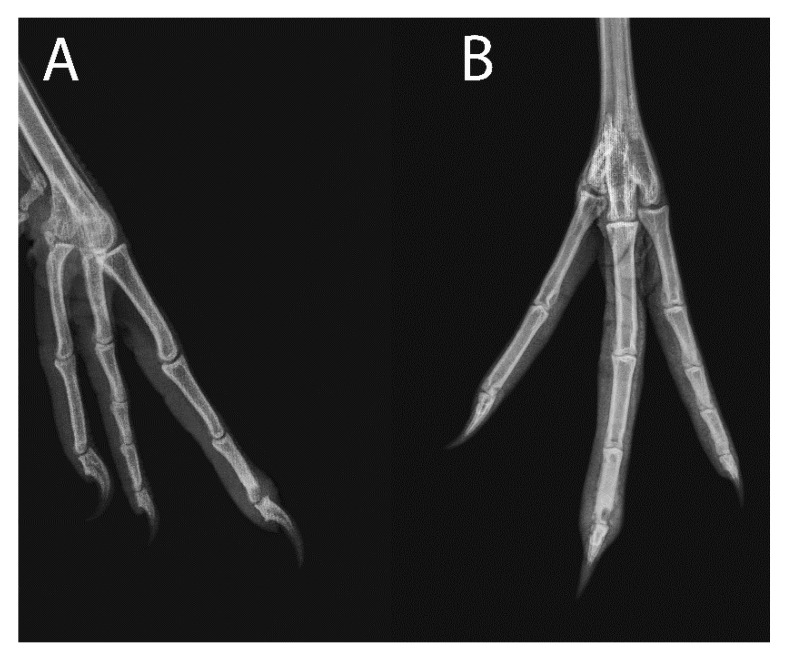
(**A)** Lateral and (**B**) dorsoplantar radiographs of the right foot of Case 2, a 31-year-old male Siberian crane (*Leucogeranus leucogeranus*) with probable osteomyelitis of the third digit of the right foot at time of presentation.

Case 2, a 31-year-old male Siberian crane (*Leucogeranus leucogeranus*), was presented for altered behavior per animal care staff. On physical exam, the third digit of the right foot was swollen with the distal interphalangeal joint markedly swollen and warm to the touch. Radiographs showed bony lysis and soft tissue swelling of the distal aspect of the third phalanx of the right third digit (Figure 2). An RLP was performed in the same manner as Case 1, but with 5 mg/kg amikacin sulfate (50 mg/mL; Wedgewood Pharmacy) and 0.5 mg/kg meloxicam (5 mg/mL; Boehringer Ingelheim Animal Health). The bird was then started on ceftiofur (Excede, Zoetis; 20 mg/kg IM q7d). On day 6, the third digit appeared unchanged, so RLP was repeated under manual restraint. Following the procedure, the bird was started on enrofloxacin (Baytril, Bayer Corp; 10 mg/kg PO q24h for 14 days) and piroxicam (Greenstone LLC, Peapack, NJ 07977, USA; 0.9 mg/kg PO q24 for 14 days). The third digit swelling had improved slightly and RLP was repeated on day 13 under manual restraint. On day 20, the soft tissue swelling had resolved, although the distal aspect of the third phalanx was still mildly enlarged. The bird’s lameness had also resolved and oral antibiotics and anti-inflammatories were discontinued. The digital enlargement persists 3 years following treatment with no recurrence of soft tissue swelling or lameness.

**Figure 3:**
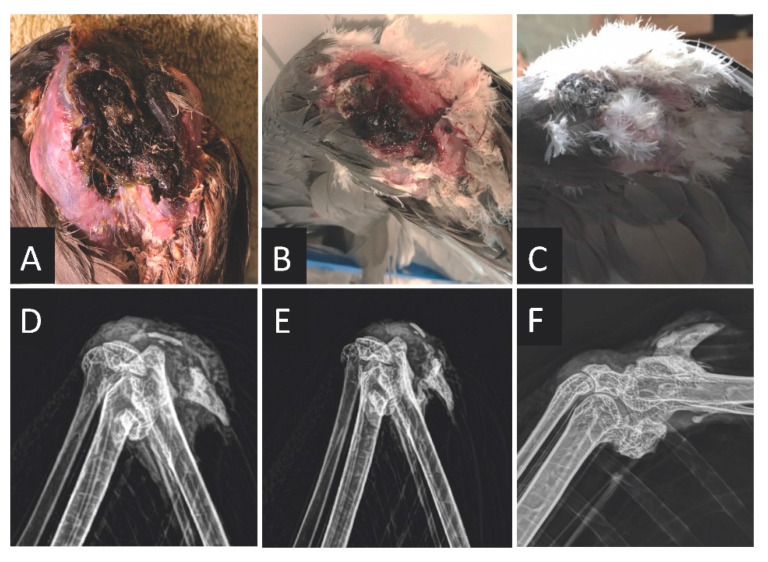
The left carpus of Case 3, an 11-year-old male wattled crane (*Bugeranus carunculatus*), with septic arthritis and suspected alular osteomyelitis at the time of presentation and after treatment with intravenous antibiotic regional limb perfusion. Palmodorsal (PD) photographs of the wound (**A**) 4 days after presentation, (**B**) 6 weeks later, and (**C**) 10 weeks after presentation. PD radiographs (**D**) at the time of presentation, (**E**) 6 weeks later, and (**F**) 16 months after presentation.

Case 3, an 11-year-old male wattled crane (*Bugeranus carunculatus*), presented with an approximately 6 cm × 3 cm scabbed wound on the ventral aspect of the left carpus that exuded synovial fluid on carpal extension. Radiographs showed potential mineralization of the soft tissue surrounding the carpus and an irregular margin and radiolucency of the left proximal alula, suggestive of osteomyelitis (Figure 3). A CBC and biochemistry panel were within reported reference intervals [8]. Ceftiofur (Excede, Zoetis) was provided at 21 mg/kg IM q4d for 2 doses. On day 4, the bird was anesthetized with isoflurane and a fluctuant swelling was noted on the palmar aspect of the carpus. Carpal arthrocentesis yielded cloudy synovial fluid; cytological analysis showed degenerate heterophils with Gram-negative, rod-shaped bacteria. An aerobic culture of the fluid isolated *Acinetobacter* sp. and *Enterobacter* sp., both of which were resistant to beta-lactam antibiotics and susceptible to fluoroquinolones and aminoglycosides (Supplementary Culture Report S1). Regional limb perfusion was performed on the left wing by manually occluding the left ulnar vein approximately 2 cm proximal to the elbow against the underlying bone on the ventral aspect of the wing, and serial infusion of the following: amikacin sulfate (Amiglyde-V, 250 mg/mL, Zoetis; 10 mg/kg IV), flunixin meglumine (Banamine, 50 mg/mL, Merck Animal Health, Summit, NJ 07901, USA; 1.25 mg/kg IV), and saline with a 23-ga butterfly catheter and allowed to infuse for 15 min. Hemostasis of the ulnar vein was achieved through manual pressure with gauze for approximately 1 min following RLP. No leakage from the vessel was confirmed visually. Consideration was given to using a penrose drain as a tourniquet around the proximal wing, but the presence of the patagial ligament was thought to prohibit adequate occlusion. Saline at 62.5 mL/kg SQ was administered following the procedure. Based on culture and sensitivity results, ceftiofur was discontinued and oral marbofloxacin was started (Zeniquin, Zoetis; 75 mg PO q24h × 40 days). On day 9, the swelling on the dorsal aspect of the carpus was subjectively reduced and the animal had normal left wing carriage. On day 23, prolific granulation tissue was present over the wound, and synovial fluid could still be expressed from it. A second RLP procedure was performed with amikacin sulfate (Amiglyde-V, 250 mg/mL; 10 mg/kg IV) followed by saline 3 mL IV in the same manner as the first procedure, but without flunixin meglumine. After cleaning with dilute chlorhexidine (MWI Animal Health, Boise, ID 83705, USA) and application of topical gentamicin/betamethasone (GenOne Spray, MWI Animal Health), the wound was then covered with a non-adherent dressing and transparent film dressing (Tegaderm Film 3M Healthcare, St. Paul, MN 55144, USA). A recheck exam on day 30 showed no swelling around the left carpal joint and the wound had healthy underlying granulation tissue with serosanguinous discharge. A repeat biochemistry panel showed the uric acid was still within reference range [8]. On day 51, the left carpal wound was significantly smaller in size (approximately 2.5 cm × 1.5 cm) with no soft tissue swelling or expressible synovial fluid on palpation. Radiographs performed under general anesthesia with isoflurane showed sharper demarcation and resolution of radiolucency of the proximal alula. A repeat biochemistry panel was within reported reference intervals [8]. Oral marbofloxacin therapy was discontinued and a recheck examination on day 81 showed almost complete healing of the left carpal wound with a small, superficial scab and feather regrowth. Sixteen months post-treatment, this bird had no recurrence of the left carpal wound or osteomyelitis, radiographs showed good remodeling and resolution of the irregular alular margins, and a biochemistry panel was within reported reference intervals [8].

## 3. Discussion

To date, there are no published reports of the use of RLP in cranes and this the first report of intravenous RLP in a wing of a bird. While manual occlusion of the ulnar vein was used in Case 3, the procedure could likely be performed using a tourniquet over the proximal wing as well. Previous reports in avian species have largely focused on infections of the distal hindlimb (cellulitis, osteomyelitis, tenosynovitis) and treatment with amikacin and ampicillin/sulbactam have resulted in resolution of these infections in combination with systemic antibiotics and anti-inflammatories in brown pelicans and a rooster [4,6]. Ceftiofur RLP has been investigated experimentally in chickens, which found the synovial concentration of ceftiofur exceeded therapeutic concentrations for most pathogenic bacteria [5]. A pilot study assessing intraosseous regional limb perfusion in a chicken wing showed the amikacin concentration in the elbow did not reach expected levels, based on reports from equine RLP [7].

Culture and sensitivity results in Case 3 showed amikacin was an appropriate choice for RLP. In Cases 1 and 2, culture and sensitivity were not performed and amikacin was selected empirically. Amikacin is a frequent first line RLP antibiotic in horses [9]. Anti-inflammatory drugs have also been used in RLP in combination with antibiotics in horses and a rooster [6,10].

The medications used in RLP in these cases (amikacin, flunixin meglumine, and meloxicam) are potentially nephrotoxic in avian species when administered systemically. While amikacin is less nephrotoxic than other aminoglycosides, it has been reported to cause renal damage [11]. No negative sequelae have been noted in these birds. The International Crane Foundation (ICF) has used meloxicam in 113 cranes of all 15 species; none were associated with significant antemortem morbidity or postmortem lesions [12]. Flunixin meglumine, paired with fluid therapy, was chosen in Case 3 based on its successful use in a RLP report [6]. While there are no published studies showing adverse effects associated with the use of flunixin meglumine in cranes, historical treatments with this drug in 14 cranes led to 5 of them developing fatal visceral gout at ICF [12]. Flunixin meglumine is no longer recommended for use in this collection.

The major limitation in discussing the use of RLP in these three cases is the significance of RLP to the resolution of infection in these cases, especially since all three cases were also treated with systemic antibiotics and anti-inflammatories. This issue has also been reported in equine medicine and is unclear based on a review of RLP studies in horses [13]. Ideally, comparing the time of resolution in these three cases to another distal limb infection case with comparable systemic therapy and no RLP treatments would be a better determinant of the significance of RLP in case outcome. However, based on the authors’ experience, the cases in this report appeared to have a shorter disease course and faster time to case resolution.

## 4. Conclusions

Antibiotic intravenous RLP is a useful adjunctive therapy for distal limb infections with no adverse effects recognized in three cranes and should be considered in conjunction with systemic antibiotics and anti-inflammatories for treating unresponsive pododermatitis, cellulitis, osteomyelitis, and septic arthritis. Further research into its use as a sole therapy for treatment of distal limb bacterial infections as well as safety and dosing studies in avian species are needed.

## Data Availability

Not applicable.

## Supplementary Materials

The following are available online at https://www.mdpi.com/article/10.3390/ani11092673/s1, Culture Report S1: Anaerobic/aerobic and fungal cultures of wound fluid obtained via carpal arthrocentesis in Case 3, a wattled crane.
