# Supplementary material for: Adjunctive Use of Intravenous Antibiotic Regional Limb Perfusion in Three Cranes with Distal Limb Infections
Source: Animals (Basel). 2021 Sep 12;11(9):2673. doi: 10.3390/ani11092673 (PMC8464672; doi:10.3390/ani11092673)
Supplement: Supplementary file 1 [file animals-11-02673-s001.zip › animals-1358066-supplementary.pdf]

IDEXX

MALE WATTLED CRANE

PET OWNER:	**WATTLED CRANE**			LAB ID:	
SPECIES:	Avian, Other			ORDER ID:	
BREED:	Crane, Wattled			COLLECTION DATE:	**10/29/18**
GENDER:	Male			DATE OF RECEIPT:	**10/30/18**
AGE:	14 Years	ACCOUNT #:		DATE OF RESULT:	**11/13/18**
PATIENT ID:		ATTENDING VET:			

IDEXX Services: **Anaerobic/Aerobic & Fungal Cultures**

Microbiology

10/30/18 (Order Received)
11/13/18 7:09 AM (Last Updated)

Source: WOUND

Culture Results:

Status: FINAL

Isolate 1: Acinetobacter sp. - 3+

Isolate 2: Enterobacter sp. - 3+

Isolate 3: Enterococcus faecalis - 1+

For Amoxicillin susceptible isolates, treatment with Amoxicillin, Ampicillin or Penicillin is recommended. Other antibiotics to which the isolate is susceptible should be considered as secondary options reserved for the treatment of Amoxicillin-resistant isolates. Although Fluoroquinolones MAY be effective in treating Enterococcal infections, in vitro susceptibility does NOT consistently predict clinical efficacy. Vancomycin should be reserved for the treatment of serious systemic infections and is NOT recommended for treatment of simple urinary tract infections. Trimethoprim/Sulfa, Clindamycin, Gentamicin and all Cephalosporins (including Cefovecin and Cefpodoxime), are NOT reported due to lack of correlation of in-vitro testing with clinical effectiveness and are not recommended for the treatment of Enterococcal infections.

	Isolate 1	MIC	Isolate 2	MIC
Tobramycin	S		S	
Amoxicillin	R	>=32	N/I	
Amoxicillin-Clavulanic Acid	I	16	R	>=32
Cephalexin	R	>=64	R	>=64
Cefpodoxime	R	>=8	R	>=8
Cefovecin	R	>=8	R	>=8
Ceftazidime	S	8	R	32
Ceftiofur	R	>=8	R	>=8
Imipenem	S	1	R	>=16
Amikacin	S	<=2	S	<=2
Gentamicin	S	<=1	S	<=1
Ciprofloxacin	S	0.12	S	<=0.06
Enrofloxacin	S	<=0.12	S	<=0.12
Marbofloxacin	S	<=0.5	S	<=0.5
Doxycycline	S	<=0.5	S	4
Chloramphenicol	R	32	I	16
Trimethoprim/Sulphate	S	<=20	S	<=20
Cefotaxime	S		S	
Erythromycin	N/I		N/I	
Minocycline	N/I		N/I	
Florfenicol	N/I		N/I	
Azithromycin	N/I		N/I	

	Isolate 3	MIC
Tobramycin	N/I	
Amoxicillin	S	
Amoxicillin-Clavulanic Acid	S	<=2
Cephalexin	N/I	
Cefpodoxime	N/I	
Cefovecin	N/I	
Ceftazidime	N/I	
Ceftiofur	N/I	

Microbiology (continued)

Imipenem	N/I	
Amikacin	N/I	
Gentamicin	N/I	
Ciprofloxacin	I	
Enrofloxacin	I	1
Marbofloxacin	I	2
Doxycycline	S	<=0.5
Chloramphenicol	S	8
Trimethoprim/Sulphate	N/I	
Cefotaxime	N/I	
Erythromycin	R	
Minocycline	S	<=0.5
Florfenicol	S	<=4
Azithromycin	R	

INTERPRETATION KEY for Antibiotic Susceptibility Results (when performed)
S = Sensitive. Organism is inhibited by usual recommended dose.
I = Intermediate. Organism is inhibited only by the maximum recommended dose.
R = Resistant. Organism is resistant to the maximum recommended dose.
These standards have been established by the Clinical and Laboratory Standards Institute (CLSI).
TF = To Follow. Susceptibility testing for this antibiotic is performed by Kirby-Bauer and results will follow shortly.

N/I (not indicated) will be reported and/or MIC data may be left blank and not reported if:
a) the growth requirements of the organism require the sensitivity testing to be performed by another method
b) interpretive criteria are not available from CLSI (in this case, recommended antibiotics will be reported based on clinical efficacy studies)
c) certain antibiotics are not available due to limitations of our commercial laboratory system; or
d/ the drug is known to be clinically ineffective against the organism regardless of in vitro results
If "N/I" is listed for ALL antibiotics for a specific isolate, susceptibility testing was not performed for that organism. Please refer to the comment associated with the organism for recommendations if applicable.

For more information on Minimum Inhibitory Concentration (MIC) please see the "Microbiology Guide to Interpreting Minimum Inhibitory Concentration (MIC)" section of the IDEXX Reference Laboratories Directory of Services or visit www.idexx.com/MIC.

Anaerobic Results:

Status: FINAL

No organisms isolated anaerobically

Microbiology

10/30/18 (Order Received)
11/13/18 7:09 AM (Last Updated)

Source: WOUND

Fungal Culture Results:

Status: FINAL

NO FUNGAL GROWTH
